# Non‐pharmaceutical treatment options for meibomian gland dysfunction

**DOI:** 10.1111/cxo.13035

**Published:** 2020-01-13

**Authors:** Reiko Arita, Shima Fukuoka

**Affiliations:** ^1^ Itoh Clinic Saitama Japan; ^2^ Lid and Meibomian Gland Working Group Tokyo Japan; ^3^ Omiya Hamada Eye Clinic Saitama Japan

**Keywords:** intense pulsed light, intraductal probing, meibomian gland, meibomian gland dysfunction, thermal pulsation

## Abstract

This review examines currently available non‐pharmaceutical treatment modalities for meibomian gland dysfunction. A detailed search of the PubMed and MEDLINE databases was performed to identify original articles in English that have evaluated such nonpharmaceutical therapies in patients with this condition. Conventional therapies such as application of a warming compress, the practice of lid hygiene, and manual expression of meibomian glands as well as more technologically advanced approaches such as intraductal probing, thermal pulsation, and intense pulsed light therapy are included in the review. These non‐pharmaceutical treatment options may each have a role to play in the management of meibomian gland dysfunction, but more studies are necessary to compare treatments directly under identical experimental conditions in order to determine their relative efficacy. Additional large‐scale, randomised, controlled trials are also required to provide more information such as the specific indications best suited to each treatment modality, the efficacy of such approaches in combination with pharmaceutical‐based therapy, and the mechanisms of action of some of the more technologically advanced systems.

Meibomian gland dysfunction (MGD) is commonly encountered in ophthalmic clinics. However, MGD has tended to be overlooked, in part because of a large discrepancy between its signs and symptoms, because it does not cause blindness, and because until recently there was no internationally accepted consensus regarding its definition. In 2011, the International Workshop on Meibomian Gland Dysfunction convened by the Tear Film and Ocular Surface Society proposed a definition of MGD: ‘MGD is a chronic, diffuse abnormality of the meibomian glands, commonly characterised by terminal duct obstruction and/or qualitative/quantitative changes in the glandular secretion. This may result in alteration of the tear film, symptoms of eye irritation, clinically apparent inflammation, and ocular surface disease.’^2011^ Since then, various high‐tech devices have been developed for the diagnosis and treatment of MGD, potentially heralding a new era in which clinicians will be able to choose and combine treatment options depending on the severity of the condition. In this article, non‐pharmaceutical treatment options for MGD, from the long‐standing to more recently introduced, are reviewed.

## Method of literature review

In this review, we focused on non‐pharmaceutical treatments for MGD that are commonly implemented worldwide on the basis of scientific evidence provided by multiple studies performed in different regions. We selected six main types of non‐pharmaceutical treatments based on eyelid warming, lid hygiene, manual expression of meibomian glands, intraductal probing, thermal pulsation, or intense pulsed light. English language articles in the PubMed and MEDLINE databases were searched by the authors, with the last access in February 2019. A search with the key words ‘warm’, ‘eyelid’, and ‘meibomian’ for eyelid warming resulted in the selection of 57 abstracts, which, after the removal of review articles, papers not in English, and studies that did not focus on eyelid warming in MGD patients, were narrowed down to 13 full‐length articles for analysis. In a similar way, eight full‐length articles for management of lid hygiene were finally included on the basis of a search with the key words ‘lid’, ‘hygiene’, and ‘meibomian’ that yielded 47 abstracts; four full‐length articles for manual expression of meibomian glands were finally included on the basis of a search with the key words ‘digital’ and ‘meibomian’ that yielded 24 abstracts; seven full‐length articles for intraductal probing were finally included on the basis of a search with the key words ‘probing’ and ‘meibomian’ that yielded 13 abstracts; 16 full‐length articles for thermal pulsation were finally included on the basis of a search with the key words ‘thermal pulsation’ and ‘meibomian’ that yielded 21 abstracts; and 14 full‐length articles for intense pulsed light were finally included on the basis of a search with the key words ‘intense pulsed light’ and ‘meibomian’ that yielded 21 abstracts.

## Treatment modalities

### Eyelid warming

Application of a warm compress is usually the first step of treatment for MGD.^2011^ The meibum secreted by meibomian glands begins to melt at 32°C for healthy individuals but at 35°C for patients with the obstructive form of MGD.^1998^ Application of heat to the eyelids promotes melting of the altered meibum of MGD patients and consequent unplugging of the gland orifices, encouraging the oily secretion to flow unimpeded as in healthy individuals. The minimum effective temperature for this therapy was recently found to be 41.5°C.^2019^ A study of the effects of two eyelid‐warming devices (EyeGiene and Blephasteam) on the lipid composition (lipidome) of tear fluid in individuals with MGD revealed a reduction in the abundance of lysophospholipids that was associated with increased tear film stability as well as an increase in the levels of (*O*‐acyl)‐ω‐hydroxy fatty acids, a reduced rate of ocular evaporation and an improvement in ocular symptoms.^2014^ In this section, the safety and efficacy of various eyelid‐warming therapies classified according to the mechanism of warming – warm compresses, steam warming, and radiant heat – are reviewed (Table 1).

**Table 1:** Studies which have shown the safety and efficacy of eyelid warming

Study, country	Study design	Number of eyes and subjects (mean ± SD age) for warming group	Number of eyes and subjects (mean ± SD age) for control group	Main outcomes	Device	Level of evidence
Goto et al.,^2002^ Japan	Prospective, non‐comparative	37 eyes of 37 MGD patients (51 ± 21.2 years)	None	Improved ocular symptoms, TBUT, corneal and conjunctival staining, and MG obstruction score	Eye Hot	III
Mori et al.,^2003^ Japan	Prospective, non‐randomised, controlled	34 eyes of 17 MGD patients (53.8 ± 14.7 years)	Untreated 16 eyes of 8 MGD patients (53.4 ± 17.5 years)	Improved tear film stability and uniformity of the lipid layer of the tear film	Hot Eye Mask	II
Purslow,^2013^ UK	Prospective, non‐comparative	25 eyes of 25 healthy subjects (29.2 ± 5.7 years)	None	Provided sufficient warming to melt meibum and reduced hyperaemia	Blephasteam	III
Benitez Del Castillo et al.,^2014^ Spain	Prospective, non‐comparative	73 eyes of 73 MGD patients (55.3 ± 17.3 years)	None	Most patients found the device comfortable to use and were able to continue with activities such as watching television, reading, and using a computer, findings that might be expected to contribute to compliance	Blephasteam	III
Bilkhu et al.,^2014^ UK	Prospective, randomised, single‐masked, controlled	25 eyes of 25 MGD patients (28.7 ± 7.8 years)	Contralateral eyes	Device was safe and effective, with its effects persisting for up to 6 months	MGDRx EyeBag	I
Sim et al.,^2014^ Singapore	Prospective, assessor‐blinded, randomised, controlled	50 MGD patients (53.5 ± 11.1 years), with 25 each for Blephasteam and EyeGiene	25 MGD patients (56.3 ± 11.0 years) for a warm towel	Blephasteam was more effective than a warm towel and EyeGiene; each therapy was safe with regard to visual acuity for 3 months of treatment	Blephasteam, EyeGiene, warm towel	I
Villani et al.,^2015^ Italy	Prospective, non‐comparative	50 MGD patients (64 ± 12 years)	None	Improved ocular symptoms and tear film stability, and reduced acinar diameter and area as detected by *in vivo* confocal microscopy	Blephasteam	III
Wang et al.,^2015^ New Zealand	Prospective, examiner‐masked, randomised, paired‐eye	41 eyes of 41 MGD patients (26.7 ± 12.9 years)	Contralateral eyes	Both devices clinically and significantly improved NIBUT and LLG, whereas the MGDRx EyeBag was more effective at raising ocular temperature	EyeGiene, MGDRx EyeBag	I
Arita et al.,^2015^ Japan	Prospective, examiner‐masked, randomised	10 healthy subjects (32.3 ± 11.7 years) and 10 MGD patients (75.6 ± 6.7 years)	The order of devices was randomised	Dry warming was more effective for improving tear film stability and meibum condition than wet warming both in healthy subjects and MGD patients	Azuki‐no‐Chikara, Eye Hot R, Hot Eye Mask, Memoto Esthe, hot towel	I
Murakami et al.,^2015^ USA	Prospective, randomised, paired‐eye	5 eyes of 5 healthy subjects (42.2 ± 20.3 years)	Contralateral eyes	The bundle method, although the most labour‐intensive, was the most effective at increasing eyelid temperature above the therapeutic level	EyeGiene, Bruder Moist Heat Eye Compress, MGDRx EyeBag, TheraPearl mask, rice bag, bundled hot towels, Tranquileyes (Eyeeco), Blephasteam	I
Bitton et al.,^2016^ Canada	Prospective, randomised, controlled	12 healthy subjects (23.2 ± 3.8 years)	The order of devices was randomised	All devices with the exception of a hot towel showed stable heat retention over 12 minutes	MGDRx EyeBag, EyeDoctor, Bruder Moist Heat Eye Compress, Tranquileyes XR, TheraPearl, hot towel	I
Arita et al.,^2017^ Japan	Prospective, randomised, controlled, crossover	20 eyes of 20 healthy subjects (34.9 ± 6.8 years) and 36 eyes of 36 patients with dry eye (30.4 ± 5.7 years)	Crossover	Single or repeated application of a menthol‐containing heated mask significantly improved tear meniscus volume, TBUT, and meibum condition in both healthy subjects and dry eye patients	Hot Eye Mask containing menthol and similar mask without menthol	I
Turnbull et al.,^2018^ New Zealand	Prospective, single‐visit, randomised	81 eyes of 81 MGD patients (46 ± 18 years), with 25 eyes for Blephasteam and 28 eyes for MGDRx EyeBag	28 eyes for liposomal spray	All 3 treatments improved tear film quality in a manner independent of MGD severity	Blephasteam, MGDRx EyeBag, liposomal spray	I

LLG: lipid layer grade, MG: meibomian gland, MGD: meibomian gland dysfunction, NIBUT: non‐invasive break‐up time, TBUT: tear film break‐up time.

#### Warm compresses

Warm compresses include hot towels as well as microwaveable bags containing beads or seeds such as Medibeads (Bruder Healthcare, Alpharetta, GA, USA), the EyeDoctor (The Body Doctor, Huddersfield, UK), the MGDRx EyeBag (The EyeBag Company, Halifax, UK), Azuki‐no‐Chikara (Kiribai, Osaka, Japan), and many others.

The simplest approach to warm‐compress therapy is the application of a hot towel. However, this approach has not been standardised for the treatment of MGD, with patients applying the towel for various times at various temperatures and with varying degrees of compliance.^2011^ One study found that application of a hot towel at 45°C for a total of at least four minutes, with replacement of the towel with a new one at the same temperature every two minutes, resulted in eyelid warming sufficient to melt meibum in individuals with MGD.^2008^ Such a procedure is probably not realistic for the performance of warm‐compress therapy by patients at home. Although hot towels have been found to be effective for the treatment of MGD, they have also been reported to induce transient visual impairment due to corneal distortion, as evidenced by the polygonal reflex of Fischer‐Schweitzer and that apparently results from the associated application of light pressure.^6^, ^7^ Therapy with a hot towel was found to be not as effective with regard to heat retention compared with microwaveable bags containing beads or wheat.^2016^ The application of bundled hot towels allows an appropriate temperature to be maintained, although, again, compliance is unlikely to be good.^2015^

The MGDRx EyeBag is a reusable silk and cotton eye compress that contains flaxseed and which is heated in a microwave and applied by the patient to the affected eye. A randomised, examiner‐masked clinical trial of the MGDRx EyeBag for the treatment of patients with MGD revealed efficacy with regard to improvement in ocular symptoms, the non‐invasive break‐up time and lipid layer thickness of the tear film, the osmolarity of tear fluid, and meibomian gland dropout and function.^2014^ The efficacy of the MGDRx EyeBag has also been compared with that of the EyeGiene mask (see below).^11^, ^12^ Both devices resulted in a clinically and statistically significant improvement in the non‐invasive break‐up time of the tear film and lipid layer grade, as evaluated in a study of 41 patients with mild‐to‐moderate dry eye symptoms.^2015^ However, the MGDRx EyeBag was found to be more effective at raising the ocular temperature, and the temperature profile of the skin surface was more uniform and the skin cooled more slowly after heating with the MGDRx EyeBag than with the EyeGiene mask.^2015^

Azuki‐no‐Chikara consists of a bag containing red beans that is heated in a microwave oven. It was compared with another dry device, Eye Hot infrared warming goggles (Cept Co, Tokyo, Japan), three wet devices, Hot Towel (Daiso, Hiroshima, Japan), Hot Eye Mask (Kao, Tokyo, Japan), and Memoto Esthe (Panasonic, Osaka, Japan) in one study with both MGD patients and control subjects.^2015^ Among the five devices, Azuki‐no‐Chikara was found to be the most effective in addition to being reusable, patient‐friendly, and available at a reasonable cost. The bag was heated for 100 seconds in a 500‐W microwave and placed on the eyelids for five minutes twice daily. The findings of this study also suggested that dry warming was more effective, especially for patients with the obstructive form of MGD, than was wet warming, which might result in a lowering of eyelid temperature due to evaporative cooling after heat application.^2015^

#### Steam‐based devices

Blephasteam (Spectrum Thea Pharmaceuticals, Macclesfield, UK) is an electrical device consisting of a pair of goggles that generates a warm, moist environment. These goggles were found to be safe and to increase the temperature of the upper and lower eyelids in 25 normal subjects by 1.7 ± 0.9°C and 2.1 ± 0.7°C, respectively.^2013^ Subsequent studies demonstrated the safety and efficacy of Blephasteam in patients with MGD or dry eye.^15^, ^16^, ^17^, ^18^, ^19^

Hot Eye Mask, a disposable menthol‐containing warming device, was shown to improve the lipid layer of the tear film in a study with 17 MGD patients.^2003^ It was also found to increase tear volume and to soften meibum, resulting in an improvement in tear film stability, in patients with dry eye.^2017^

#### Radiant heat‐based devices

The EyeGiene (Eyedetec Medical, Danville, CA, USA) consists of a reusable eye mask that contains disposable warming units that are activated by squeezing and inserted into the mask immediately before use. The mask delivers heat at a temperature of 40°C for up to five minutes within 30–60 seconds of activation. Heat production is based on a sustained thermochemical reaction. A randomised, non‐controlled, three‐arm study comparing a hot towel (n = 10), the EyeGiene mask (n = 12), and Blephasteam (n = 10) in MGD patients found that the efficacy of each treatment was similar with regard to improvement of ocular symptoms and meibomian gland parameters.^2014^ A randomised, controlled trial of the same three approaches in 75 patients with MGD showed that Blephasteam was more effective than a hot towel for MGD treatment, with the hot towel and EyeGiene being similarly effective.^2014^ The EyeGiene has also been compared with the MGDRx EyeBag as described above.^11^, ^12^

The safety and efficacy of Eye Hot infrared warming goggles were examined in 37 patients with MGD.^2002^ The goggles were found to improve tear film stability in association with an increase in meibum release.

#### Further considerations

Studies have thus shown that eyelid‐warming therapies are generally safe and effective for the treatment of MGD (Table 1). Given that warming devices have been found to be effective in patients with or without meibomian gland dropout,^2018^ individuals at all stages of MGD should be encouraged to perform and continue eyelid warming not only for symptom relief but also to prevent further deterioration of their condition. Large‐scale, prospective, randomised studies comparing the effects of different warming devices on subjective symptoms and objective findings in healthy control subjects and MGD patients are now needed. In addition, more evidence‐based investigations are necessary to provide insight into the mechanism of action for eyelid‐warming therapy, and the development of additional novel eyelid‐warming technologies is awaited.

### Lid hygiene

Patients with MGD are often recommended to practise lid hygiene, in combination with heat application and eyelid massage, in the home setting.^2011^ Both the application of a warming compress and the practice of lid hygiene are recommended to be performed twice daily.^2011^ Patients should be instructed that, after application of a hot compress, they wash their eyelids, especially around the cilia, with lateral movement of a finger and with the use of lukewarm water. Lid scrubbing and massage were thus found to increase tear film stability in patients with MGD.^1990^ Eyelid cleaning products such as an eyelid cleanser^2018^ and lid hygiene shampoo^2019^ as well as cleaning with ofloxacin ophthalmic ointment^2017^ ameliorated ocular symptoms and reduced ocular surface inflammation in patients with blepharitis or MGD. Lid hygiene shampoo^2019^ and cleaning with ophthalmic ointment^2017^ also improved tear film stability. Cleansing eye pads were also found to improve eyelid margin status with regard to lid margin staining, meibomian gland expression, and meibomian gland blockage.^2012^ Novel products, devices, and techniques such as the use of an eye brush^2019^ for the practice of lid hygiene warrant further investigation with regard to their efficacy given the importance of lid hygiene and compliance with recommended procedures. Given that there appears to have been only one double‐masked, randomised, controlled trial of lid hygiene therapy for patients with MGD,^2018^ more of such clinical studies are necessary to confirm the efficacy of this frequently administered treatment option.

Lid hygiene is also thought to be important because of the association of *Demodex* mites with MGD.^29^, ^30^, ^31^, ^32^ Long‐term practice of lid hygiene is necessary in individuals with *Demodex* infestation as it is a chronic condition that requires chronic therapy. *Demodex folliculorum* and *Demodex brevis* are thought to be the most common ectoparasites in humans. In the eye, *D. folliculorum* is found preferentially in the lash follicles and *D. brevis* in lash sebaceous glands.^29^, ^33^ There is a strong association between ocular demodicosis and ocular surface inflammatory conditions such as blepharitis, chalazia, and keratitis as well as MGD.^29^, ^32^, ^34^, ^35^ The pathogenesis of *Demodex* infestation has remained unclear;^36^, ^37^, ^38^ however, this is in part because demodicosis has a high age‐dependent prevalence and is present frequently in asymptomatic individuals.^2010^

*Demodex* mites are resistant to a wide range of antiseptic agents, including 75% alcohol, 10% povidone‐iodine, and erythromycin.^2007^ *In vivo* microscopic observation for 150 minutes revealed that *D. folliculorum* was killed by tea tree oil in a dose‐dependent manner.^2007^ In addition to this action, tea tree oil has been found to manifest antibacterial,^2004^ antifungal,^2004^ and anti‐inflammatory^2004^ effects. Terpinen‐4‐ol, a terpene with antimicrobial, antifungal, antiviral, antiseptic, and acaricidal properties, is the active ingredient of tea tree oil with regard to the killing of *Demodex* mites.^43^, ^44^ A concentration of tea tree oil as low as 5% applied to the lids and base of the eyelash follicles twice daily or as high as 50% applied once weekly was found to attenuate *Demodex* infestation.^39^, ^44^ However, tea tree oil can cause dermatitis, allergy, and ocular irritation, especially at higher concentrations,^2012^ and treatment to eradicate *Demodex* completely is often unrealistic.

### Manual expression of meibomian glands

**Figure 1:**
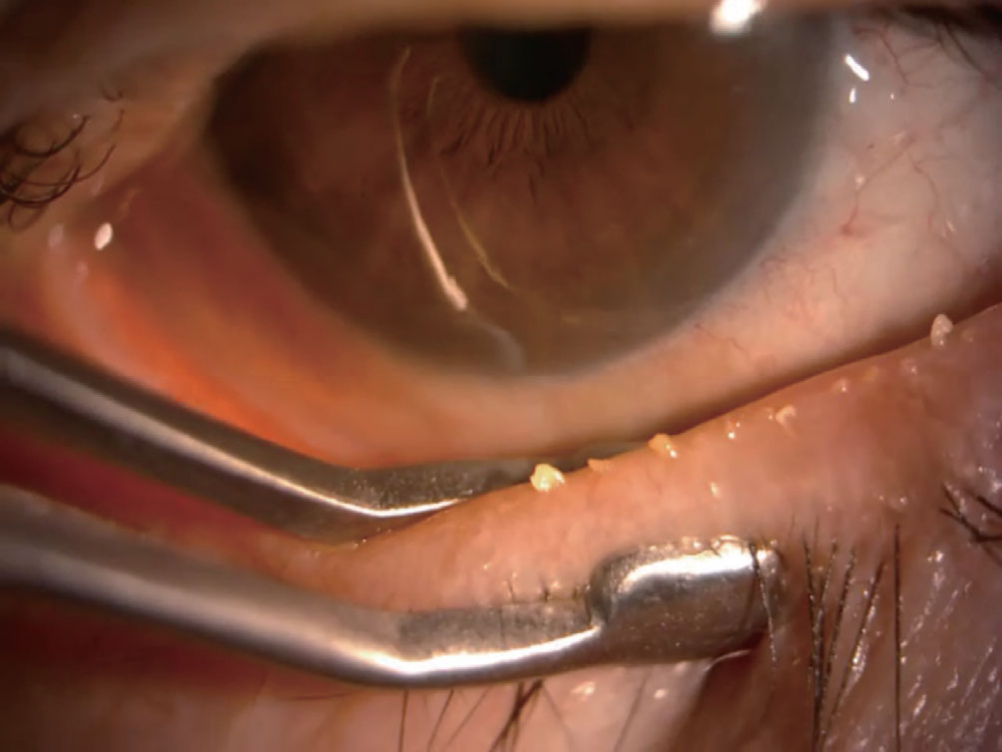
Therapeutic expression of thickened and toothpaste‐like meibum in a patient with meibomian gland dysfunction with the use of an Arita meibomian gland compressor

Physical expression of meibomian glands for therapeutic purposes is an in‐office procedure with a history of at least 90 years.^2^, ^46^, ^47^, ^48^, ^49^ It is achieved by forceful squeezing of the eyelids either against each other or between a rigid object (such as a glass rod, cotton swab, or metal paddle) on the inner lid surface and a finger, thumb, or other rigid object on the outer surface (Figure 1).^2011^ The amount of force required to express obstructed glands can be substantial and is usually limited by the associated pain.^2011^ However, in spite of the pain, several studies have demonstrated the efficacy of manual meibomian gland expression for the treatment of MGD.^51^, ^52^, ^53^ It is recommended that the procedure be performed once a month until the gland dysfunction is resolved.^2011^ Although expression of meibomian glands appears to be safe and effective, its evaluation by observer‐masked, randomised, controlled trials is necessary, as is the development of new technologies that can achieve such expression with reduced pain.

### Intraductal probing

Intraductal probing of meibomian glands with a microcannula is performed as an in‐office procedure to open gland orifices and offer symptomatic relief to patients with obstructive MGD (Table 2).^2010^ This procedure, conducted with the aid of a slitlamp microscope, involves passing a 2‐mm probe directly into the orifice of each gland so as to allow egress of meibum.^2010^ Although it is relatively safe, the procedure is invasive and can cause lid haemorrhage, and some patients need more than one treatment. Probing was shown to improve the Ocular Surface Disease Index (OSDI) score at one and six months after, compared with before treatment in 10 patients with refractory MGD and ocular rosacea.^2012^ Meibomian gland lipid levels were also found to be increased by intraductal probing in three patients with refractory obstructive MGD in Japan.^2015^ A randomised, controlled study of 25 patients with obstructive MGD showed that both signs and symptoms were improved one month after probing.^2016^ Other studies have demonstrated the safety and efficacy of probing for patients with obstructive MGD.^58^, ^59^, ^60^ Moreover, probing was associated with an increase in meibomian gland area^2018^ as determined by non‐invasive meibography.^2013^

**Table 2:** Studies which have shown the safety and efficacy of intraductal probing

Study, country	Study design	Number of eyes and subjects (mean ± SD age) for probing group	Number of eyes and subjects (mean ± SD age) for control group	Main outcomes	Level of evidence
Maskin,^2010^ USA	Prospective, non‐randomised, non‐controlled	25 obstructive MGD patients (70.2, 37–93 years)	None	Probing was safe and ameliorated ocular symptoms including lid tenderness	III
Nakayama et al.,^2015^ Japan	Prospective case study	6 lid margins of 3 refractory MGD patients (age not listed)	None	Probing improved meibum lipid levels as measured with a meibometer as well as reduced meibum viscosity	III
Ma and Lu,^2016^ China	Prospective, randomised, controlled	25 MGD patients (57.7 ± 11.2 years)	24 MGD patients (55.5 ± 10.6 years) treated with 0.1% fluorometholone	Probing improved subjective symptoms, meibum grade, TBUT, lid margin abnormalities, and fluorescein staining compared with the control	I
Sik Sarman et al.,^2016^ Turkey	Prospective, non‐randomised, non‐controlled	58 eyes of 30 refractory MGD patients (47 ± 4.5 years)	None	Probing improved ocular symptoms (OSDI) and TBUT for up to 3 months as well as reduced hyperaemia and lid margin vascularity	III
Syed and Sutula,^2017^ USA	Retrospective	70 eyelids of 40 refractory MGD patients (57.4, 27–92 years)	None	Dynamic intraductal probing ameliorated ocular symptoms and was safe for treatment of refractory obstructive MGD	III
Maskin and Testa,^2018^ USA	Retrospective	34 eyelids of 19 patients (62.3 ± 13.3 years)	None	Probing increased total gland area and mean individual gland area observed by non‐contact meibography	III
Incekalan et al.,^2019^ Turkey	Prospective, randomised, controlled	40 eyes of 20 MGD patients (51.8 ± 12.9 years)	40 eyes of 20 MGD patients (52.2 ± 11.5 years) receiving conventional treatment	Probing induced rapid symptom relief and clinical improvement	I

MGD: meibomian gland dysfunction, OSDI: Ocular Surface Disease Index, TBUT: tear film break‐up time.

Of note, neither subjects nor investigators were masked to treatment allocation in the studies of intraductal probing performed to date (Table 2), indicating that caution should be exercised in drawing conclusions from their findings. The follow‐up periods of the studies were also relatively short, with the result that data on the long‐term safety and efficacy of this invasive technique are lacking. In addition, in most studies, probing was not compared with standard treatments in the clinical setting such as eyelid warming, lid hygiene, or meibomian gland expression. Further studies without potential bias are thus necessary to confirm the safety and efficacy of this procedure.

### Thermal pulsation system

**Figure 2:**
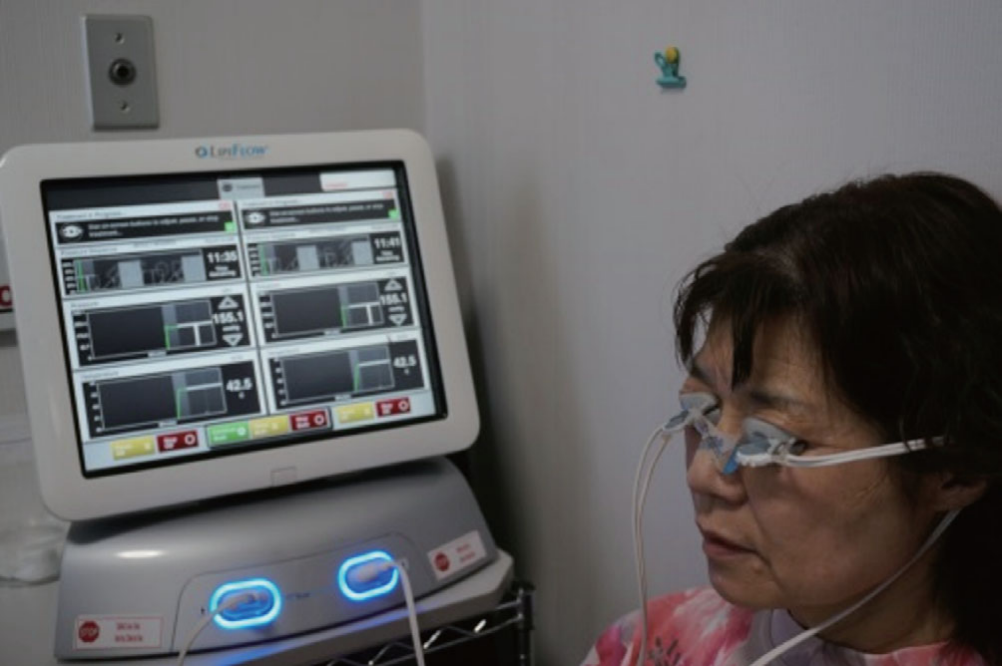
Thermal pulsation system. A LipiFlow thermal pulsation system (Johnson & Johnson Vision) is applied bilaterally to a 65‐year‐old woman with mild meibomian gland dysfunction.

The LipiFlow Vectored Thermal Pulsation (VTP) System (Johnson & Johnson Vision, Jacksonville, FL, USA) is an automated thermodynamic device for in‐office treatment of MGD (Figure 2). The device applies direct heat (42.5°C) to the palpebral conjunctiva of the upper and lower eyelids directly over the meibomian glands in order to soften meibum, while simultaneously applying pulsatile pressure to the outer eyelids with an inflatable air bladder that compresses the glands.^63^, ^64^ Case studies^63^, ^65^, ^66^ as well as controlled^18^, ^64^, ^67^, ^68^, ^69^, ^70^, ^71^, ^72^ and non‐controlled^73^, ^74^, ^75^, ^76^, ^77^ trials have shown that a single 12‐minute VTP therapy can improve meibomian gland function, ocular surface staining, and tear film break‐up time as well as relieve ocular symptoms (Table 3). Such improvement in meibomian gland function and dry eye symptoms achieved with a single VTP therapy session can be sustained for one to three years.^68^, ^74^, ^77^ The improvement in meibomian gland function induced by VTP therapy has been evaluated on the basis of the number of meibomian glands yielding liquid secretion (MGYLS) in the lower eyelid^63^, ^66^, ^67^, ^68^, ^69^, ^70^, ^72^, ^75^, ^76^, ^77^ or the meibomian gland secretion (MGS) score,^64^, ^65^, ^68^, ^71^, ^73^, ^74^, ^75^, ^77^ which represents both the number of secreting meibomian glands and the quality of the secreted material as determined with a meibomian gland evaluator (MGE, Johnson & Johnson Vision). Some prospective trials have found that the lipid layer thickness of the tear film as determined with a LipiView interferometer (Johnson & Johnson Vision) did not change significantly at an average of 52 days or greater than three months after a single VTP therapy.^69^, ^70^, ^76^ On the other hand, one prospective, randomised trial demonstrated an improvement in lipid layer thickness at six months after VTP therapy.^2014^ A single VTP session was shown to be at least as effective at improving subjective symptoms as was either application of a warm compress daily for two weeks^2012^ or twice daily for three months^67^, ^68^, ^69^ or oral administration of doxycyclin twice daily for three months.^2018^ Prospective, randomised, controlled studies have also found that a single VTP therapy improved the MGS to a greater extent compared with a warm compress^64^, ^68^ and was more effective at reducing the conjunctival tear evaporation rate than was EyeGiene, Blephasteam, or a warm towel.^2016^ On the other hand, some prospective, controlled studies have found that a single VTP session was equivalent in its improvement of meibomian gland function to twice daily administration either of a warm compress^2014^ or of oral doxycycline^2018^ for three months.

**Table 3:** Studies which have shown the safety and efficacy of thermal pulsation (VTP system)

Study, country	Study design	Number of eyes and subjects (mean ± SD age) in the VTP group	Number of eyes and subjects (mean ± SD age) in the control group	Main outcomes	Level of evidence
Korb and Blackie,^2010^ USA	Prospective case study	Both eyes of 1 MGD patient (39 years)	None	VTP increased TBUT and the number of functional MGs as well as reduced ocular symptoms for up to 3 months	III
Lane et al.,^2012^ USA	Prospective, randomised, controlled, crossover, multicentre	138 eyes of 69 MGD patients (age not listed)	140 eyes of 70 MGD patients (age not listed) treated with a warm compress, with crossover to VTP	VTP was more effective than a warm compress applied daily for 2 weeks	I
Greiner,^2012^ USA	Prospective, non‐randomised, non‐controlled, multicentre	42 eyes of 21 MGD patients (62.2 ± 12.1 years)	None	VTP improved MG secretion, TBUT, and ocular symptoms for up to 9 months	III
Korb and Blackie,^2013^ USA	Prospective case study (the same patient as in Korb and Blackie [2010])	Both eyes of 1 MGD patient (39 years)	None	VTP improved MG secretion and reduced symptoms for up to 7 months in a patient with severe MG atrophy	III
Greiner,^2013^ USA	Prospective, non‐randomised, non‐controlled	36 eyes of 18 MGD patients (63.2 ± 12.1 years)	None	VTP increased the number of functional MGs and reduced symptoms for up to 1 year	III
Finis et al.,^2014^ Germany	Prospective, randomised, controlled, crossover	17 eyes of 17 MGD patients (45 ± 23 years)	14 eyes of 14 MGD patients (50 ± 19 years) treated with lid warming and massage, with crossover to VTP	VTP improved the number of functional MGs and symptoms and was as effective as lid hygiene practised twice daily for 3 months	I
Finis et al.,^2014^ Germany	Prospective, non‐controlled (including patients who participated in the trial by Finis et al. [2014] above)	52 eyes of 26 MGD patients (50 ± 22 years); VTP only (n = 17), VTP after lid hygiene for 3 months (n = 9)	None	VTP improved MG function and symptoms at 6 months, but had no effect on MG atrophy; patients with severe MG atrophy responded poorly	III
Satjawatcharaphong et al.,^2015^ USA	Prospective, non‐randomised, non‐controlled	64 eyes of 32 MGD patients (54.4 ± 15.0 years), including those with hypersecretory MGD	None	Severity of baseline symptoms and male gender were associated with symptomatic improvement after VTP	III
Greiner,^2016^ USA	Prospective, non‐randomised, non‐controlled	40 eyes of 20 MGD patients (61.4 ± 11.2 years); subcohort of the original study by Lane et al. (2012)	None	VTP improved MG secretion, the number of functional MGs, and symptoms for up to 3 years	III
Yeo et al.,^2016^ Singapore	Prospective, randomised controlled	24 eyes of 24 MGD patients (70.0 ± 16.0 years)	22 eyes of 22 MGD patients in each of three groups treated with a hot towel, EyeGiene, or Blephasteam twice daily (67.0 ± 21.7, 57.7 ± 22.7, and 69.7 ± 22.6 years, respectively)	VTP reduced conjunctival tear evaporation rate at 3 months and was more effective than a warm towel	I
Blackie et al.,^2016^ USA	Prospective, crossover, multicentre	188 eyes of 99 MGD patients (56.2 ± 15.3 years)	196 eyes of 98 MGD patients treated with a warm compress and lid hygiene, with crossover to VTP	VTP improved MG secretion and reduced symptoms over 1 year; early VTP for MGD was associated with improved treatment outcomes	I
Zhao et al.,^2016^ Singapore	Prospective, controlled, non‐randomised	25 eyes of 25 MGD patients (55.6 ± 12.7 years)	25 eyes of 25 MGD patients (56.4 ± 11.4 years) treated with a warm compress	VTP was as effective as twice daily application of a warm compress for 3 months; treatment efficacy was not affected by pre‐treatment MG loss	II
Zhao et al.,^2016^ China	Prospective, contralateral eye	29 eyes of 29 MGD patients (56.90 ± 7.07 years)	Contralateral eye (the eye the patient perceived as less affected)	Monocular VTP improved the number of functional MGs and symptoms compared with the control eye for up to 3 months	II
Kenrick and Alloo,^2017^ USA	Prospective case study	Right eye of 1 patient (28 years)	Bruder Moist Heat Eye Compress, Blephasteam, and MiBoFlo Thermaflo before VTP	VTP increased the temperature of the inner surface of the eyelid to the 40°C therapeutic threshold for melting of obstructive meibum	III
Epitropoulos et al.,^2017^ USA	Retrospective, controlled	43 eyes of 23 MGD patients with SS (62 ± 13.8 years)	59 eyes of 36 MGD patients without SS	The improvement in MG secretion at 2 months after VTP was smaller in MGD patients with SS than in those without SS	II
Hagen et al.,^2018^ USA	Prospective, randomised, parallel‐group	26 eyes of 13 MGD patients (51.7 ± 15.6 years)	24 eyes of 12 MGD patients (50.4 ± 14.4 years) treated with oral doxycycline	VTP improved the signs of MGD and was as effective as oral doxycycline administration for 3 months	I

MG: meibomian gland, MGD: meibomian gland dysfunction, SS: Sjögren's syndrome, TBUT: tear film break‐up time, VTP: vectored thermal pulsation.

None of the studies of VTP have been double‐masked, and so the placebo effect may have influenced any perceived improvement in subjective symptoms. However, no study has found that VTP therapy was less effective than other treatments. VTP treatment thus appears to be safe and effective, and its principle based on warming and compression of meibomian glands without pain seems sound. Given that MGD is a chronic disease, the long‐term efficacy of VTP needs to be evaluated further.

### Intense pulsed light

**Figure 3:**
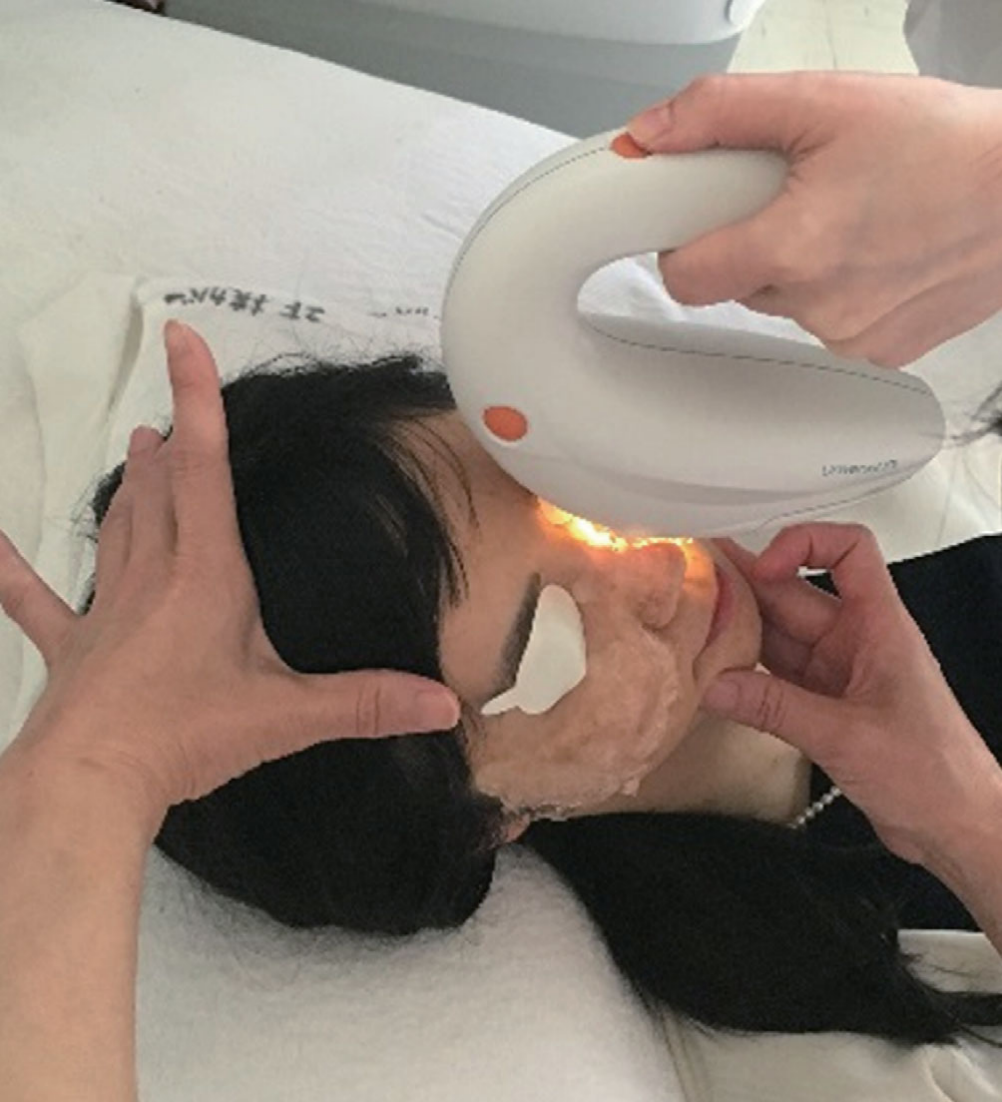
Intense pulsed light therapy is administered with an M22 system (Lumenis) to a 38‐year‐old woman with moderate meibomian gland dysfunction

Intense pulsed light (IPL) therapy is administered during an office visit with devices such as Lumenis OPT M22 (Lumenis, Yokneam, Israel), E‐Eye (E‐Swin, Gambais, France), and DermaMed Quadra 4 IPL (DermaMed, Lenni, PA, USA) (Figure 3). The devices deliver high‐intensity visible light from a broad‐spectrum (wavelength of 500 to 1,200 nm), noncoherent, polychromatic light source. Light at these wavelengths can excite melanin and haemoglobin in skin and thereby induce coagulation and ablation of blood vessels.^2008^ IPL therapy is generally administered for the treatment of dermatologic conditions, but an early clinical study of its application to patients with MGD indicated that it may be beneficial for amelioration of the signs and symptoms of this condition (such as lid erythema and telangiectasia), with dermatologic adverse effects being apparent in up to 13 per cent of individuals.^2015^ More than 10 subsequent studies have demonstrated the safety and efficacy of IPL for the treatment of MGD (Table 4).^80^, ^81^, ^82^, ^83^, ^84^, ^85^, ^86^, ^87^, ^88^, ^89^, ^90^, ^91^, ^92^ A double‐masked, placebo‐controlled, paired‐eye study showed that IPL can improve lipid quality and quantity in MGD patients.^2015^ A prospective and randomised study found that a series of treatment sessions including both IPL and meibomian gland expression resulted in a significant improvement in subjective symptoms and objective signs compared with gland expression alone in patients with refractory MGD.^2019^ This study evaluated 12 parameters including those related to meibomian glands and the lipid layer of the tear film both before treatment as well as after each of the eight treatment sessions and for up to 11 weeks after the final session.^2019^ The results thus indicated that IPL plus meibomian gland expression is a promising therapeutic approach for patients with refractory MGD. With regard to the possible mechanism underlying the therapeutic action of IPL in MGD patients, the treatment was found to significantly reduce the levels of inflammatory markers such as interleukin‐17A and interleukin‐6 in tear fluid of such patients.^2017^ A recent study of IPL also showed that it changed the lipid profile of meibum in MGD patients.^2019^

**Table 4:** Studies which have shown the safety and efficacy of intense pulsed light (IPL)

Study, country	Study design	Number of eyes and subjects (mean ± SD age) in the IPL group	Number of eyes and subjects (mean ± SD age) in the control group	Main outcomes	Level of evidence
Toyos et al.,^2015^ USA	Retrospective, non‐randomised, non‐controlled	182 eyes of 91 dry eye patients (21–84 years)	None	IPL plus MGX was safe and effective for MGD treatment, improving ocular symptoms and TBUT	III
Craig et al.,^2015^ New Zealand	Prospective, randomised, double‐masked, placebo‐controlled, paired‐eye	28 eyes of 28 MGD patients (45 ± 15 years)	Contralateral eye	IPL was effective for MGD treatment, improving tear film quality and reducing symptoms of dry eye	I
Gupta et al.,^2016^ USA	Prospective, non‐randomised, non‐controlled, multicentre	100 MGD or dry eye patients (63, 32–92 years)	None	IPL plus MGX improved lid margin vascularity, MG secretion, TBUT, and ocular symptoms (OSDI)	III
Vegunta et al.,^2016^ USA	Retrospective	81 dry eye patients (61, 20–84 years)	None	IPL plus MGX improved MG secretion and ocular symptoms (SPEED score)	III
Jiang et al.,^2016^ China	Prospective, non‐randomised, non‐controlled	40 eyes of 40 MGD patients (63.2 ± 12.1 years)	None	IPL improved ocular symptoms, TBUT, TMH, corneal staining, lid margin abnormalities, and meibum secretion	III
Dell et al.,^2017^ USA	Prospective, non‐randomised, non‐controlled, multicentre	80 eyes of 40 MGD patients (57.5 ± 15.1 years)	None	IPL plus MGX improved ocular symptoms (SPEED score), TBUT, corneal staining, and MG secretion	III
Liu et al.,^2017^ China	Prospective, randomised, controlled, double‐masked	44 MGD patients (46.3 ± 16.9, 23–86 years)	Contralateral eye	IPL plus MGX improved ocular symptoms (SPEED score) and TBUT as well as reduced the levels of inflammatory markers in tear fluid	I
Albietz and Schmid,^2018^ Australia	Prospective, non‐randomised, non‐controlled	26 moderate‐to‐severe MGD patients (21–82 years)	None	IPL plus MGX improved meibum expressibility and quality, TBUT, corneal staining, as well as lid margin, bulbar, and limbal redness	III
Rong et al.,^2018^ China	Prospective, randomised, double‐masked, controlled	44 MGD patients (46.3 ± 16.9 years)	Contralateral eye	IPL plus MGX improved ocular symptoms (SPEED score), TBUT, and meibum secretion relative to baseline; changes in MGYLS and TBUT were greater in the study eyes than in the control eyes, but changes in SPEED and corneal staining scores were similar	I
Rong et al.,^2018^ China	Prospective, randomised, double‐masked, controlled	28 MGD patients (42.1 ± 17.6 years)	Contralateral eye	IPL plus MGX increased MG secretion and TBUT at 6 months after treatment	I
Seo et al.,^2018^ South Korea	Prospective, non‐randomised non‐controlled	17 patients with rosacea and moderate or severe MGD (64, 57–68 years)	None	IPL plus MGX improved lid margin vascularity, meibum expressibility and quality in the lower eyelid, and ocular symptoms (OSDI) for up to 12 months after treatment	III
Arita et al.,^2018^ Japan	Prospective, non‐randomised, non‐controlled, multicentre	62 eyes of 31 refractory MGD patients (47.6 ± 16.8 years)	None	IPL plus MGX improved ocular symptoms (SPEED score), NIBUT, lipid layer condition, meibum grade, lid margin abnormality scores, TBUT, and corneal staining	III
Arita et al.,^2019^ Japan	Prospective, randomised, controlled	22 refractory MGD patients (61.0 ± 18.0, 23–81 years)	20 refractory MGD patients (61.9 ± 12.2, 39–78 years) undergoing MGX alone	IPL plus MGX improved ocular symptoms (SPEED score), TFLLT, NIBUT, TBUT, lipid layer condition, lid margin abnormalities, corneal staining, and meibum grade compared with the control	I
Ahmed et al.,^2019^ Egypt	Prospective, non‐randomised, controlled	24 eyes of 12 MGD patients (50 ± 10 years)	24 eyes of 12 healthy subjects (50 ± 10 years)	IPL increased the molecular weights of lysozyme, lactoferrin, and albumin as well as the concentrations of total lipids, triglycerides, cholesterol, and phospholipids in tear fluid	II

MG: meibomian gland, MGD: meibomian gland dysfunction, MGX: meibomian gland expression, MGYLS: number of meibomian glands yielding liquid secretion, NIBUT: non‐invasive break‐up time, OSDI: Ocular Surface Disease Index, SPEED: Standard Patient Evaluation of Eye Dryness, TBUT: tear film break‐up time, TFLLT: tear film lipid layer thickness, TMH: tear meniscus height.

Among all of the studies covered in this review (Tables 1, 2, 3, 4), the evidence levels of four studies^80^, ^85^, ^87^, ^88^ regarding the safety and efficacy of IPL are among the highest. The results of these four randomised, controlled, double‐masked clinical trials suggest that IPL is a potential standard treatment for MGD from early to refractory stages. However, future studies are warranted to clarify the mechanism underlying the therapeutic efficacy of IPL for MGD.

## Conclusion

MGD is a multifactorial condition that is commonly encountered in eye clinics. Whereas many patients with MGD are initially prescribed conservative therapy consisting of the application of a warm compress and the practice of lid hygiene, a substantial proportion of these individuals do not achieve a sufficient improvement in their symptoms and signs due to a lack of treatment compliance. However, the evidence presented in this review indicates that daily eyelid warming or practice of lid hygiene is safe and effective for the treatment of MGD. Clinicians should thus encourage patients to be more vigilant in their adherence to these approaches. Manual techniques such as meibomian gland expression and intraductal probing as well as more advanced technological devices including thermal pulsation and IPL systems have the potential to ameliorate the signs and symptoms of MGD, although use of the latter systems may be cost‐prohibitive. Multicentre, randomised, controlled, non‐sponsored clinical trials with large numbers of patients are needed to establish the long‐term effectiveness of such non‐pharmaceutical options for MGD therapy. Further studies are also needed to provide information such as the specific indications best suited to each non‐pharmaceutical treatment modality, the efficacy of such approaches in combination with pharmaceutical‐based therapy, and the mechanisms of action of some of the more technologically advanced systems.
